# What Do Infrequent Goldmann Applanation Tonometry Measurements Reveal About Intraocular Pressure Profiles After Nonpenetrating Glaucoma Surgery?

**DOI:** 10.1167/tvst.15.8.28

**Published:** 2026-08-27

**Authors:** Colya N. Englisch, André M. Trouvain, Philip Wakili, H. Burkhard Dick, Kaweh Mansouri, Esther M. Hoffmann, Marc J. Mackert, Karl T. Boden, Peter Szurman

**Affiliations:** 1 Eye Clinic Sulzbach, Knappschaft Hospitals Saar, Sulzbach/Saar, Germany; 2 Department of Experimental Ophthalmology, Saarland University, Homburg/Saar, Germany; 3 Knappschaftskrankenhaus Eye Clinic, University Hospital of the Ruhr University Bochum, Bochum, Germany; 4 Swiss Visio Glaucoma Research Centre, Montchoisi Clinic, Lausanne, Switzerland; 5 Department of Ophthalmology, University of Colorado, Denver, CO, USA; 6 Department of Ophthalmology, University Medical Centre of the Johannes Gutenberg University Mainz, Mainz, Germany; 7 Department of Ophthalmology, LMU University Hospital, LMU Munich, Munich, Germany; 8 Klaus Heimann Eye Research Institute (KHERI), Sulzbach/Saar, Germany

**Keywords:** glaucoma, intraocular pressure, goldmann applanation tonometry, EyeMate, telemetry

## Abstract

**Purpose:**

To evaluate how reliably single Goldmann applanation tonometry (GAT) measurements reflect the preceding month's intraocular pressure (IOP) profile in operated open-angle glaucoma.

**Methods:**

This longitudinal study included 24 patients with a suprachoroidal telemetric IOP sensor analyzing 156 in-hospital GAT measurements, 154 simultaneous telemetric readings, and 13,734 at-home measurements across follow-up visits. Telemetric data from the 30 days prior to each visit were stratified by time of day and matched to corresponding GAT measurements, which, in turn, were ranked relative to the interquartile range (IQR) of the preceding at-home measurements. Peak-and-trough analyses were performed using hourly aggregated data.

**Results:**

The GAT and telemetric IOP measurements showed good agreement (mean ΔIOP = −0.1 ± 3.0 mm Hg). However, 59.7% of the GAT-measured IOP values exceeded the IQR, and 14.3% fell below the IQR of the corresponding telemetric distributions; only 26.0% were within the IQR. Deviations from the telemetric median were 4.2 ± 2.4 mm Hg (above IQR), −2.3 ± 1.5 mm Hg (below IQR), and 0.5 ± 1.2 mm Hg (within IQR). Of the 99 statistically significant extrema (45 peaks and 54 troughs), most peaks occurred within 3 hours before GAT measurements. Troughs were observed both before and after GAT, but with larger nyctohemeral misalignment to GAT.

**Conclusions:**

Single GAT measurements frequently failed to represent the underlying 30-day IOP profile and often missed clinically relevant peaks and troughs, despite good pointwise agreement with telemetric measurements.

**Translational Relevance:**

High-frequency telemetric monitoring provides a more comprehensive assessment of IOP dynamics and may improve individualized glaucoma management compared with infrequent GAT snapshot measurements.

## Introduction

Glaucoma is a leading cause of irreversible blindness worldwide, with intraocular pressure (IOP) representing the principal modifiable risk factor for disease progression.^1^^–^^4^ Accordingly, glaucoma management relies on lowering or modulating the IOP through medical, laser, or surgical interventions; therefore, high-frequency and reliable IOP monitoring is mandatory for optimal therapeutic decision-making. Goldmann applanation tonometry (GAT) remains the reference standard for IOP assessment.^5^ However, its use is largely restricted to clinical settings, resulting in measurements typically obtained at intervals of weeks to months. This limitation is particularly relevant because IOP is inherently dynamic and subject to fluctuations across multiple time scales, including instantaneous, diurnal, short-term (days to weeks), and long-term (months to years) variations.^6^^–^^11^ A previous study demonstrated that 69% of glaucoma eyes exhibit IOP peaks outside routine clinic hours, with fluctuations of 10 mm Hg or more within 24 hours.^12^

Although nyctohemeral fluctuations can be assessed using 24-hour GAT monitoring, such protocols are laborious and thus rarely repeated in clinical practice. Capturing short- and long-term fluctuations, which requires high-frequency measurements over extended periods, is even more challenging.^13^ However, this is clinically relevant, as numerous studies have demonstrated associations between IOP fluctuations and glaucoma progression.^6^^,^^14^^–^^23^

In this context, telemetric IOP-monitoring devices enabling high-frequency, longitudinal data acquisition have gained increasing importance.^24^ Recently, we introduced a miniaturized telemetric IOP sensor designed for implantation in the suprachoroidal space during nonpenetrating glaucoma surgery (NPGS).^25^^–^^30^ Using a dataset comprised of more than 100,000 measurements, we demonstrated that, in successfully operated patients, short-term IOP fluctuations are moderate, ranging from 1.5 to 2.0 mm Hg over 30-day intervals.^31^ These findings raise an important clinical question: To what extent do infrequent, single-point GAT measurements reflect the underlying IOP profile over extended periods? This question is particularly relevant in patients undergoing routine follow-up visits at intervals of several weeks or longer.

Highlighting the importance of high-frequency IOP monitoring for fast, precise, and adaptive therapeutic decision making in glaucoma management, the present study aimed to evaluate the diagnostic representativeness of infrequent GAT measurements in patients with open-angle glaucoma (OAG) following NPGS combined with the implantation of a telemetric IOP sensor.

## Methods

### Subjects and Study Design

This longitudinal observational analysis of at-home telemetric and in-hospital IOP measurements obtained over repeated follow-up visits is based on the patient cohort derived from the 1-year prospective, open-label, single-arm, multicenter observational ARGOS-SC01 study and its 2-year follow-up study, ARGOS-SC01_FU.^25^^–^^27^ The study included 24 patients with OAG who underwent NPGS combined with implantation of a miniaturized telemetric suprachoroidal IOP sensor (EyeMate-SC, G-Metrics GmbH, Hanover, Germany). Detailed inclusion and exclusion criteria have been described previously.^25^^–^^27^ Written informed consent was obtained from all participants. The study adhered to the tenets of the Declaration of Helsinki and was approved by the local Institutional Review Board (Ethikkommission bei der Ärztekammer des Saarlandes, ID: CIV-18-07-025065; approval no. 141/18).

### Intraocular Pressure Recordings

In-hospital GAT and telemetric EyeMate-SC measurements were simultaneously obtained at scheduled study visits on postoperative days 90, 180, 270, 360, 540, 720, 900, and 1080. GAT-measured IOP values were calculated as the average of two consecutive measurements. If the difference between these exceeded 2 mm Hg, a third measurement was performed, and the average of all three measurements was used. Between study visits, patients were instructed to perform at least four at-home telemetric IOP measurements per day, without specific guidance regarding timing. For each study visit, all at-home telemetric IOP measurements recorded during the 30 days prior to the visit were collected. Measurements obtained after the visit were not considered to avoid potential confounding due to potential treatment adjustments or interventions following the clinical assessment.

### Device

The EyeMate-SC is a microelectromechanical IOP sensor designed for permanent implantation in the suprachoroidal space. The device performs 10 IOP measurements within ≈ 1 second and reports their average.

Measurements are initiated by the patient using an external handheld reader positioned near the eye, and the result is displayed immediately. The reader can store up to 3000 measurements, which can be transmitted wirelessly to the physician. Further details regarding the technical design and surgical implantation have been described previously.^25^^–^^27^

### Data Preprocessing and Matching

Repeated at-home telemetric IOP measurements obtained within a 10-minute interval for the same patient were aggregated by calculating their mean to avoid overrepresentation of clustered measurements. For the deviation analysis, telemetric measurements were categorized into predefined time-of-day (TOD) intervals: from midnight to 2:59 AM, 3:00 AM to 5:59 AM, 6:00 AM to 8:59 AM, 9:00 AM to 11:59 AM, noon to 2:59 PM, 3:00 PM to 5:59 PM, 6:00 PM to 8:59 PM, and 9:00 PM to 11:59 PM. For each study visit, at-home telemetric IOP measurements recorded during the preceding 30 days were matched to the corresponding GAT measurement by selecting only those values obtained within the same TOD interval in which the GAT measurement had been performed. This approach ensured comparability by accounting for nyctohemeral variation.

### Deviation Analysis

To evaluate how well single GAT measurements reflected the IOP profile over time, the telemetric IOP measurements recorded at home during the 30 days prior to each visit were used to construct patient-specific IOP distributions within the corresponding TOD interval. The ranking of each GAT measurement within this distribution was then determined, as described in the Statistical Analysis section. The relationship between the ΔIOP between the GAT measurements and the mean nyctohemeral telemetric IOP measured at home over the 30 preceding days and GAT-measured IOP was investigated.

### Peak-and-Trough Analysis

To assess the ability of GAT to capture extreme IOP values, telemetric data were analyzed on an hourly basis to identify peak and trough periods within each 30-day observation window. The ΔIOP as well as the temporal relationship with regard to nyctohemeral alignment between these extremes and the corresponding GAT measurement were evaluated. The statistical approach used for this analysis is described in the Statistical Analysis section.

### Statistical Analysis

Descriptive statistics are presented as mean ± standard deviation (SD) for normally distributed variables and as median with interquartile range (IQR) where appropriate. For agreement analysis between the in-hospital GAT and simultaneous telemetric EyeMate-SC measurements, a Bland–Altman analysis was performed, including the calculation of ΔIOP (EyeMate-SC − GAT) and 95% limits of agreement (LoA).

For deviation analysis, the distribution of GAT measurements relative to the IQR of the corresponding TOD-specific telemetric measurements was analyzed descriptively. The relationship between the ΔIOP between the GAT measurements and the mean nyctohemeral telemetric IOP measured at home over the 30 preceding days and GAT-measured IOP was evaluated using a univariable linear regression model. *Z*-score normalization was performed at the patient level to account for interindividual variability while preserving all observations. Briefly, this transformation centers the data at a mean of 0 and SD of 1. Each data point was adjusted by subtracting the overall mean and then dividing by the SD.

For peak-and-trough analysis, cumulative hourly grouping of the telemetric IOP data (instead of using 3-hour TOD intervals as for deviation analysis) allowed identification of the hourly minimum and maximum IOP within each 30-day observation window. The hour with the highest observed maximum IOP was defined as the candidate peak hour, whereas the hour with the lowest observed minimum IOP was defined as the candidate trough hour. Welch's two-sample *t-*tests were used to determine whether the IOP values recorded during the identified candidate peak or trough hour differed significantly from those recorded during all other non-peak and non-trough hours. Individualized extreme IOP values (significant peaks and troughs, *P* < 0.05) were ranked in relation to GAT measurements. Given the exploratory nature of the peak-and-trough analysis, no adjustment for multiple comparisons was applied. Statistical tests were two sided, where *P* < 0.05 was considered statistically significant. Statistical analyses were performed using Python 3.8.18 and SciPy 1.14.1 (https://scipy.org/citing-scipy/).

## Results

**Figure 1.**
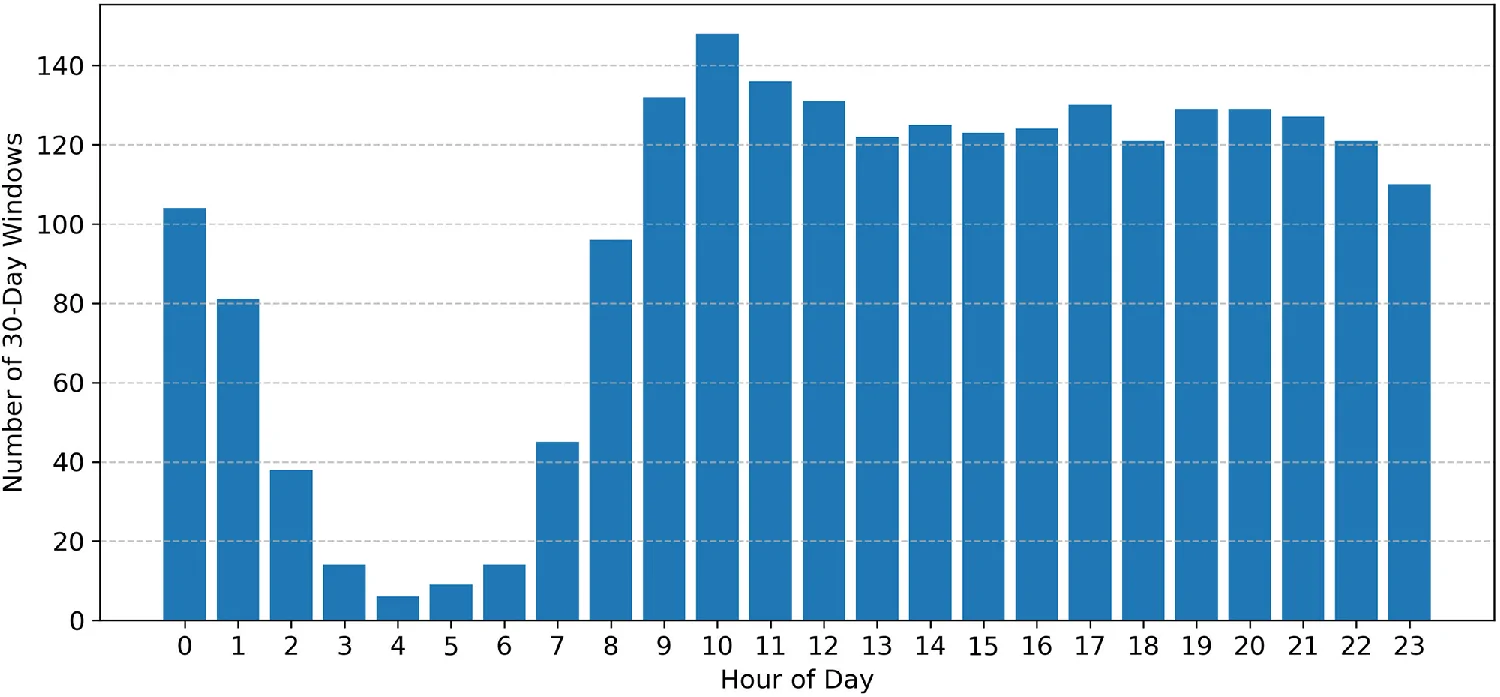
Distribution of hourly intervals (out of 24) with at least one at-home telemetric IOP measurement for each of the 154 30-day observation windows.

A total of 156 GAT measurements, 154 in-hospital telemetric measurements, and 13,734 at-home telemetric measurements were included in the analysis. GAT measurements were distributed across four of eight (possible) TOD intervals, with 101 measurements performed between 9 AM and 11:59 AM, 35 between noon and 2:59 PM, 16 between 3 PM and 5:59 PM, and two between 6 AM and 8:59 AM. On average, 88.0 ± 35.5 telemetric measurements were obtained per 30-day period. Telemetric measurements were distributed relatively homogeneously across daytime hours (Fig. 1).

### Agreement Analysis

**Figure 2.**
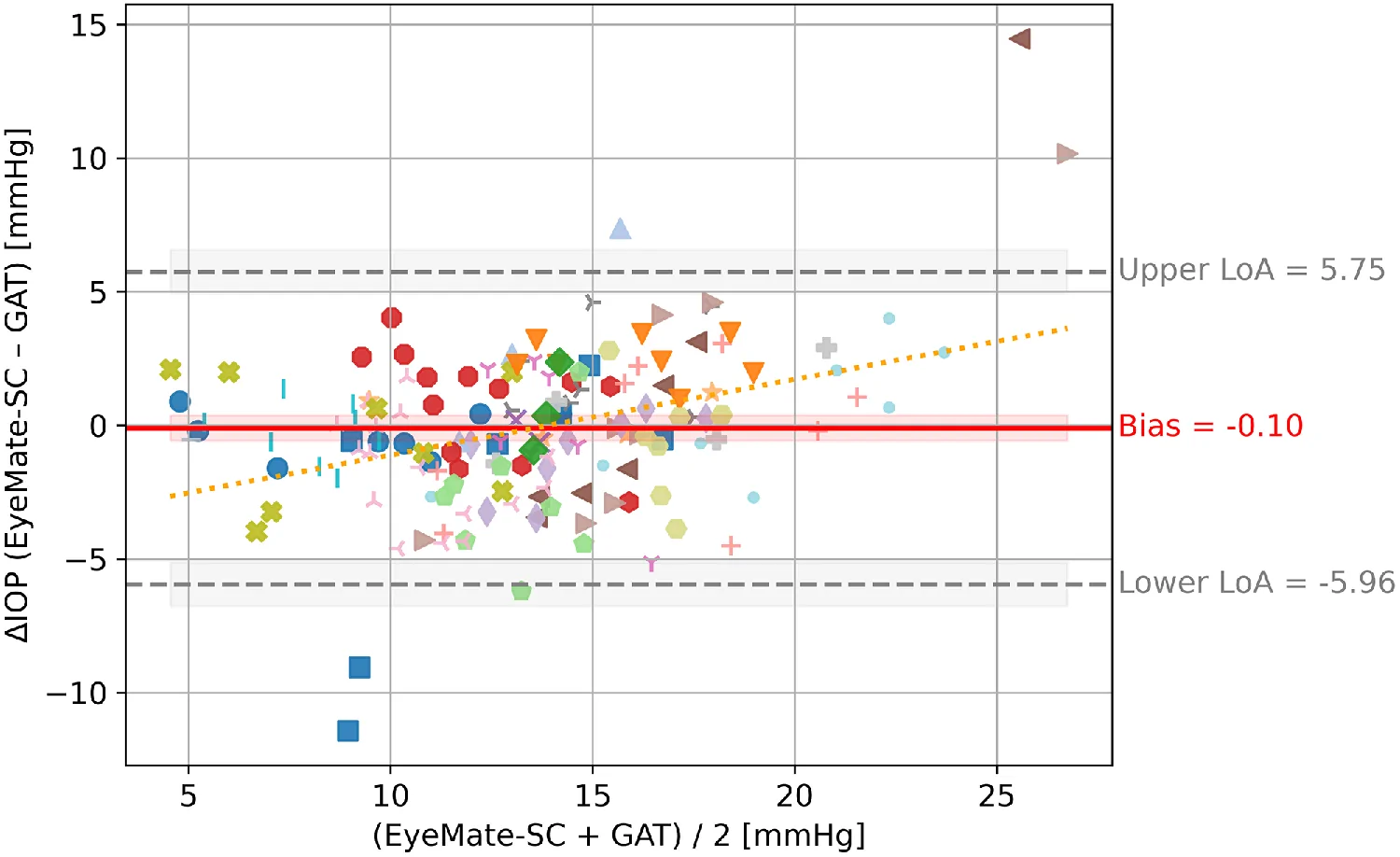
Bland–Altman plot comparing simultaneous in-hospital GAT and EyeMate-SC telemetric IOP measurements. The *x*-axis represents the mean of the GAT and EyeMate-SC measurements (mm Hg), and the *y*-axis shows the ΔIOP (EyeMate-SC – GAT; mm Hg). The *red line* indicates the mean bias (ΔIOP) with its 95% CI (*red-shaded area*), and the *gray* *lines* represent the upper and lower LoA. The *gray-shaded* area illustrates the 95% CIs of these limits. The *orange line* shows proportional bias, which represents a systematic relationship between the measurement error and the magnitude of the measurement (slope = 0.3, *P* <0.01). Each symbol represents one visit.

Simultaneous in-hospital measurements showed good agreement between GAT (13.6 ± 3.7 mm Hg) and EyeMate-SC (13.5 ± 4.8 mm Hg), with a mean ΔIOP (EyeMate-SC – GAT) of –0.1 ± 3.0 mm Hg (95% confidence interval [CI], –0.6 to 0.4 mm Hg; *n* = 154). The lower and upper LoA were –6.0 mm Hg (95% CI, –6.8 to –5.1 mm Hg) and 5.8 mm Hg (95% CI, 4.9 to 6.6 mm Hg), respectively (Fig. 2).

### Deviation Analysis

**Figure 3.**
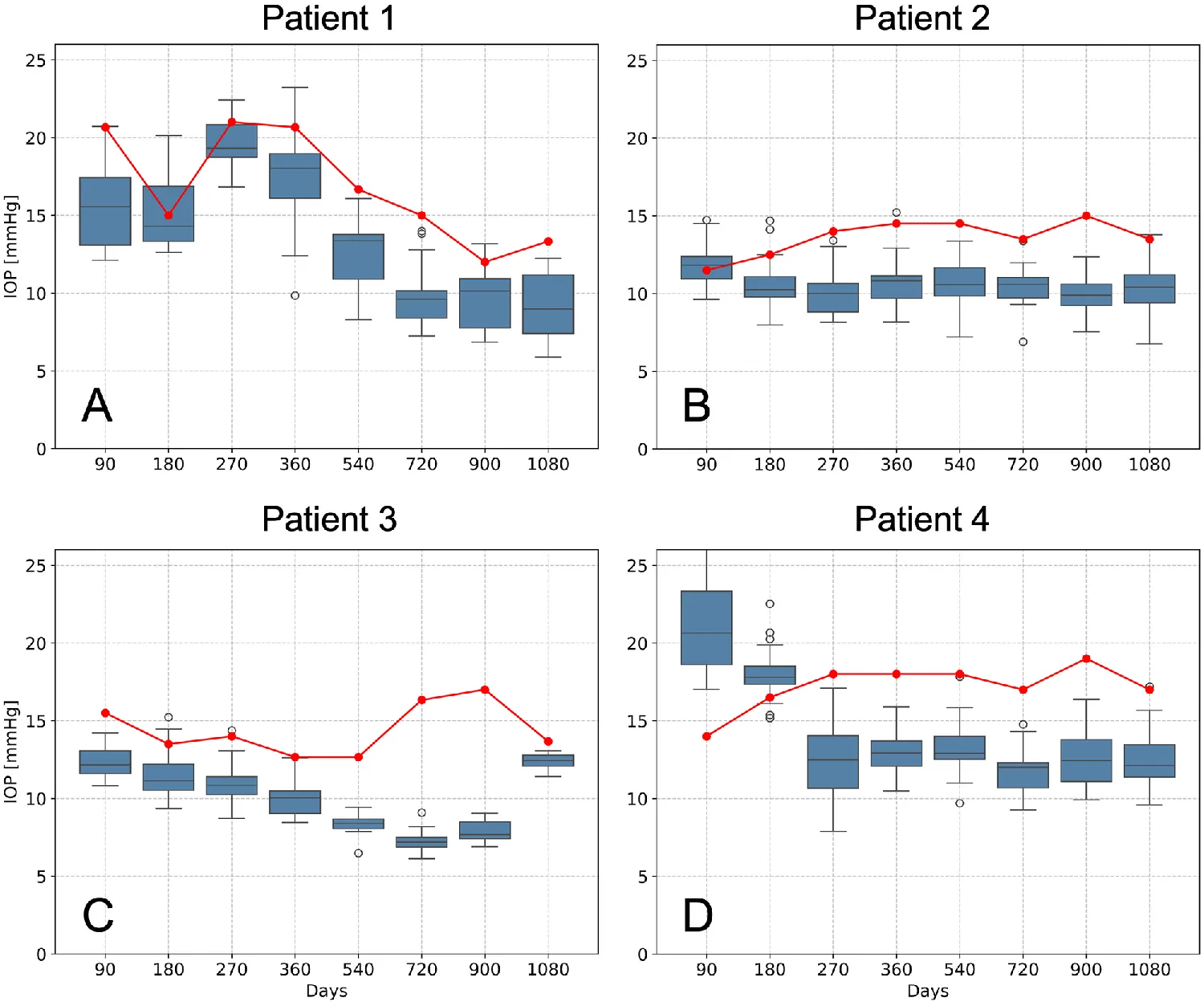
(**A**–**D**) Box plots displaying the ranking of infrequently repeated one-time GAT (*red dots*) at a clinic visit in relation to cumulative telemetric measurements (*blue boxes*) recorded over the 30 preceding days at the same TOD period in four illustrative patients throughout a time frame of 900 days. Twenty, 30, 6, 28, 30, 27, 30, and 29 (**A**); 24, 28, 24, 26, 24, 13, 10, and 11 (**B**); 16, 11, 9, 13, 9, 12, 12, and 15 (**C**); and 18, 7, 16, 4, 14, 10, 17, and 18 (**D**) telemetric measurements were obtained in the same TOD of the 30 days preceding GAT at the 90, 180, 270, 360, 540, 720, 900, and 1080 postoperative days visits. *Boxes* indicate the IQRs and medians. *Whiskers* represent the minimum and maximum values, and the individual *white dots* denote outliers.

Figure 3 shows exemplary individual relationships between the GAT and telemetric at-home IOP measurements. Although the GAT measurements and corresponding 30-day telemetric distributions of one patient were in relatively good agreement (Fig. 3A), those of the other patients showed substantial discrepancies, including opposing trends (Figs. 3B–D). Overall, the GAT measurements were higher than the upper boundary of the IQR of the corresponding telemetric distribution in 59.7% of cases. In 14.3% of cases, the GAT values were lower than the lower IQR boundary. In the remaining 26.0% of cases, the GAT measurements fell within the IQR.

The ΔIOP relative to the median of the self-measured telemetric IOP amounted to 4.2 ± 2.4 mm Hg for higher GAT values and –2.3 ± 1.5 mm Hg for lower values, and measurements within the IQR deviated by 0.5 ± 1.2 mm Hg. Table 1 summarizes the relationships between both the in-hospital GAT and concurrent telemetric measurements with the at-home self-measurements for the overall cohort and across the four relevant TOD intervals.

**Table 1.** Distribution of In-Hospital GAT (*n* = 154) and Corresponding EyeMate-SC (*n* = 154) IOP Readings Relative to the IQR of At-Home Telemetric IOP Measurements Self-Recorded Over the 30 Days Prior to Each Study Visit

Instrument	Group	Metric	Total	6–8:59 AM	9–11:59 AM	Noon–2:59 PM	3–5:59 PM
In-hospital GAT	Lower than at-home IQR	*n* (%)	22 (14.3)	0 (0.0)	14 (13.9)	4 (11.4)	4 (25.0)
		Mean ± SD	10.0 ± 3.6	—	9.6 ± 3.9	12.3 ± 2.4	9.1 ± 3.5
		Δ to median	−2.3 ± 1.5	—	−2.2 ± 1.4	−2.2 ± 2.2	−2.4 ± 1.4
	Within at-home IQR	*n* (%)	40 (26.0)	0 (0.0)	24 (23.8)	10 (28.6)	6 (37.5)
		Mean ± SD	13.0 ± 3.3	—	13.0 ± 3.5	12.9 ± 3.0	13.1 ± 3.2
		Δ to median	0.5 ± 1.2	—	0.5 ± 1.1	0.5 ± 1.5	0.8 ± 0.7
	Higher than at-home IQR	*n* (%)	92 (59.7)	2 (100.0)	63 (62.4)	21 (60.0)	6 (37.5)
		Mean ± SD	14.7 ± 3.3	18.5 ± 3.1	14.2 ± 3.3	15.2 ± 2.7	17.7 ± 3.2
		Δ to median	4.2 ± 2.4	8.2 ± 1.7	3.9 ± 2.3	4.6 ± 2.3	3.9 ± 1.9
In-hospital EyeMate-SC tonometry	Lower than at-home IQR	*n* (%)	11 (7.1)	0 (0.0)	6 (5.9)	1 (2.9)	4 (25.0)
		Mean ± SD	8.4 ± 4.2	—	7.3 ± 2.3	15.4	8.4 ± 5.6
		Δ to median	−2.4 ± 1.2	—	−2.1 ± 1.0	−3.0	−2.8 ± 1.7
	Within at-home IQR	*n* (%)	53 (34.4)	0 (0.0)	40 (39.6)	8 (22.9)	5 (31.2)
		Mean ± SD	10.9 ± 3.1	—	10.4 ± 3.1	12.6 ± 2.1	11.8 ± 3.3
		Δ to median	0.4 ± 0.7	—	0.3 ± 0.7	0.6 ± 0.8	0.5 ± 0.6
	Higher than at-home IQR	*n* (%)	90 (58.4)	2 (100.0)	55 (54.5)	26 (74.3)	7 (43.8)
		Mean ± SD	15.7 ± 4.4	13.2 ± 4.3	15.9 ± 4.6	14.5 ± 3.0	19.2 ± 6.4
		Δ to Median	3.8 ± 2.5	2.8 ± 0.5	4.0 ± 2.6	3.4 ± 1.7	4.8 ± 3.8

For each category (below, within, or above the IQR), the mean ± SD of the in-hospital measurements and the mean difference (Δ) from the median of the at-home self-measurements are reported. Data are additionally stratified by TOD of in-hospital assessment.

**Figure 4.**
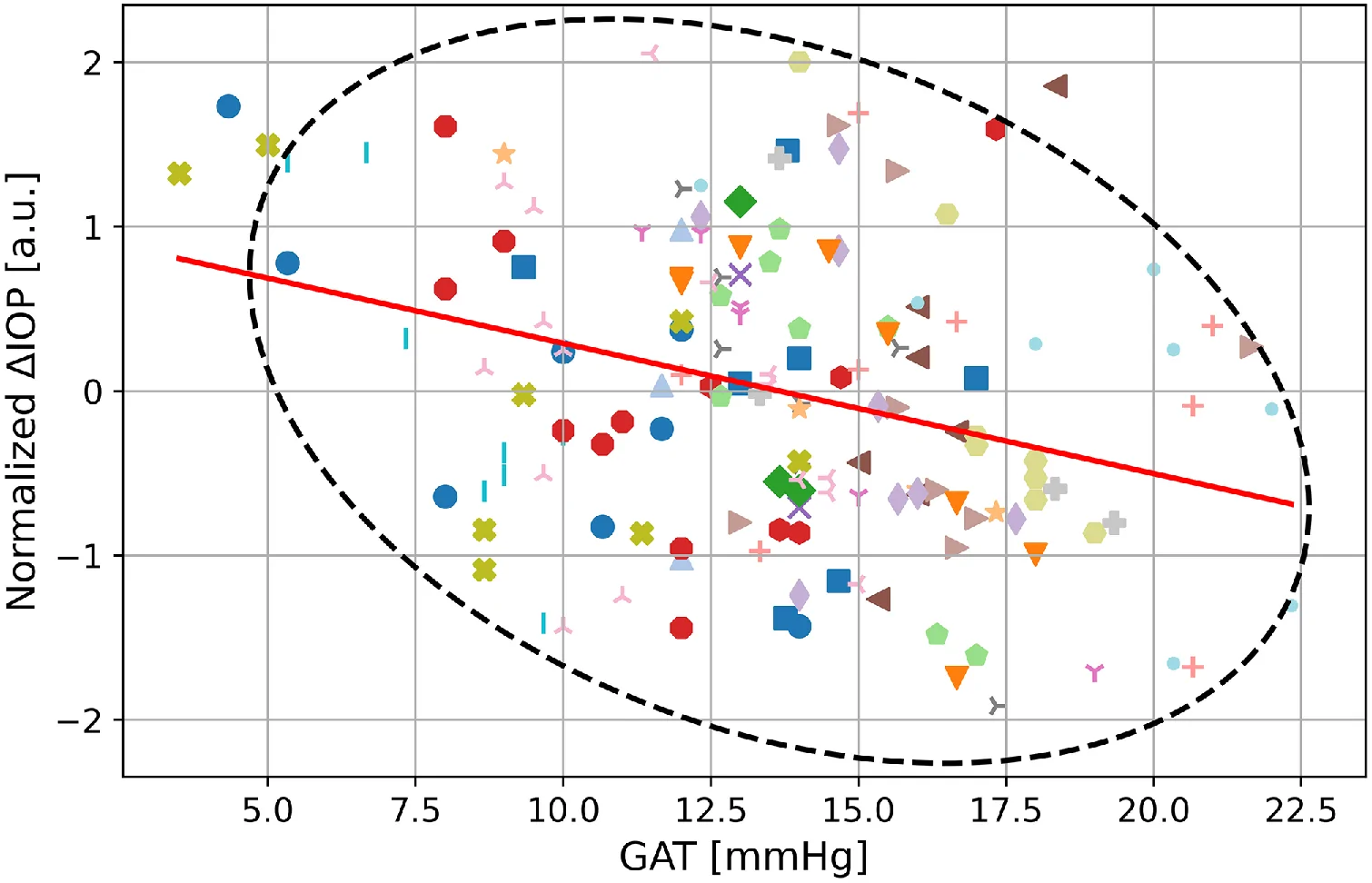
Linear regression of the ΔIOP between GAT values and the mean of the previous 30 days of at-home telemetric IOP measurements versus GAT values. The *x*-axis shows the GAT values (mm Hg); the *y*-axis shows the *z*-score–normalized ΔIOP (multipliers of the standard deviation, in arbitrary units). The *red line* shows the regression, and the ellipse represents the 95% CI around the regression. Each symbol represents one visit.

Linear regression analysis demonstrated that higher GAT-measured IOP values were significantly associated with lower patient-normalized ΔIOP between the GAT-measured IOP and 30-day mean telemetric IOP (β = –0.0815 per mm Hg; 95% CI, –0.119 to –0.044; *P* <0.001) (Fig. 4). The mean patient-specific SD of the ΔIOP was 2.60 ± 1.13 mm Hg (median ± IQR, 2.11 ± 1.68–3.41 mm Hg; range, 1.19–4.73). The model explained 10.6% of the variance (*R*^2^ = 0.106). Supplementary Tables S1 to S4 list the respective raw data.

### Peak-and-Trough Analysis

**Figure 5.**
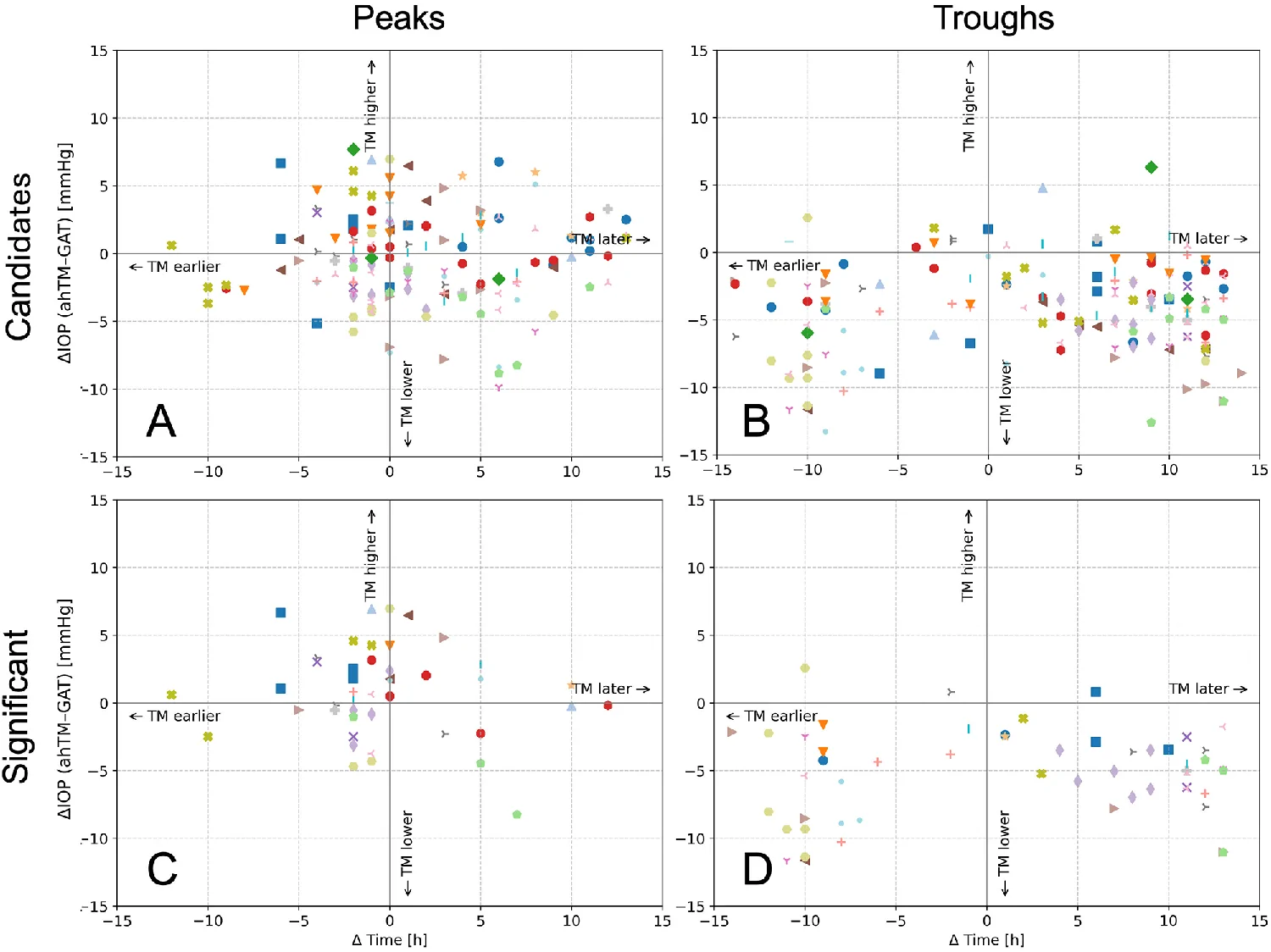
Scatterplots illustrating time- and IOP-related deviations of candidate and significant extreme hourly averaged at-home telemetric IOP measurements (ahTM), recorded during the 30 days prior to each visit, from GAT measurements. (**A**) Candidate peaks (*n* = 154), (**B**) candidate troughs (*n* = 154), (**C**) statistically significant peaks (*n* = 45), and (**D**) statistically significant troughs (*n* = 54), each relative to the corresponding GAT measurement. Each symbol represents an individual (candidate or significant) extreme IOP value relative to its matched GAT reference (origin). The *x*-axis indicates the Δtime between the telemetric extreme (peak or trough) and GAT measurement (hours), and the *y*-axis indicates the ΔIOP between the telemetric and GAT-measured IOP values (mm Hg).

A total of 154 peak-hour candidates and 154 trough-hour candidates were identified (Figs. 5A, 5B). Of these, 45 peaks and 54 troughs were statistically significant (Figs. 5C, 5D); among them, 40 peaks and 28 troughs were within 5 mm Hg of the corresponding GAT measurement. Among the statistically significant peaks, 26 occurred before (on nyctohemeral alignment) GAT measurements (Δ*t* = –3.1 ± 2.8 hours). Of these, 14 peaks were associated with higher telemetric IOP than GAT-measured IOP (ΔIOP = 2.8 ± 2.2 mm Hg), and 12 were lower (ΔIOP = –2.0 ± 1.6 mm Hg). Thirteen peaks emerged after GAT measurements (Δ*t* = 5.4 ± 3.5 h); seven were higher (ΔIOP = 3.1 ± 1.9 mm Hg) and six were lower (ΔIOP = –2.9 ± 3.0 mm Hg) than GAT-measured IOP (Fig. 5C).

**Figure 6.**
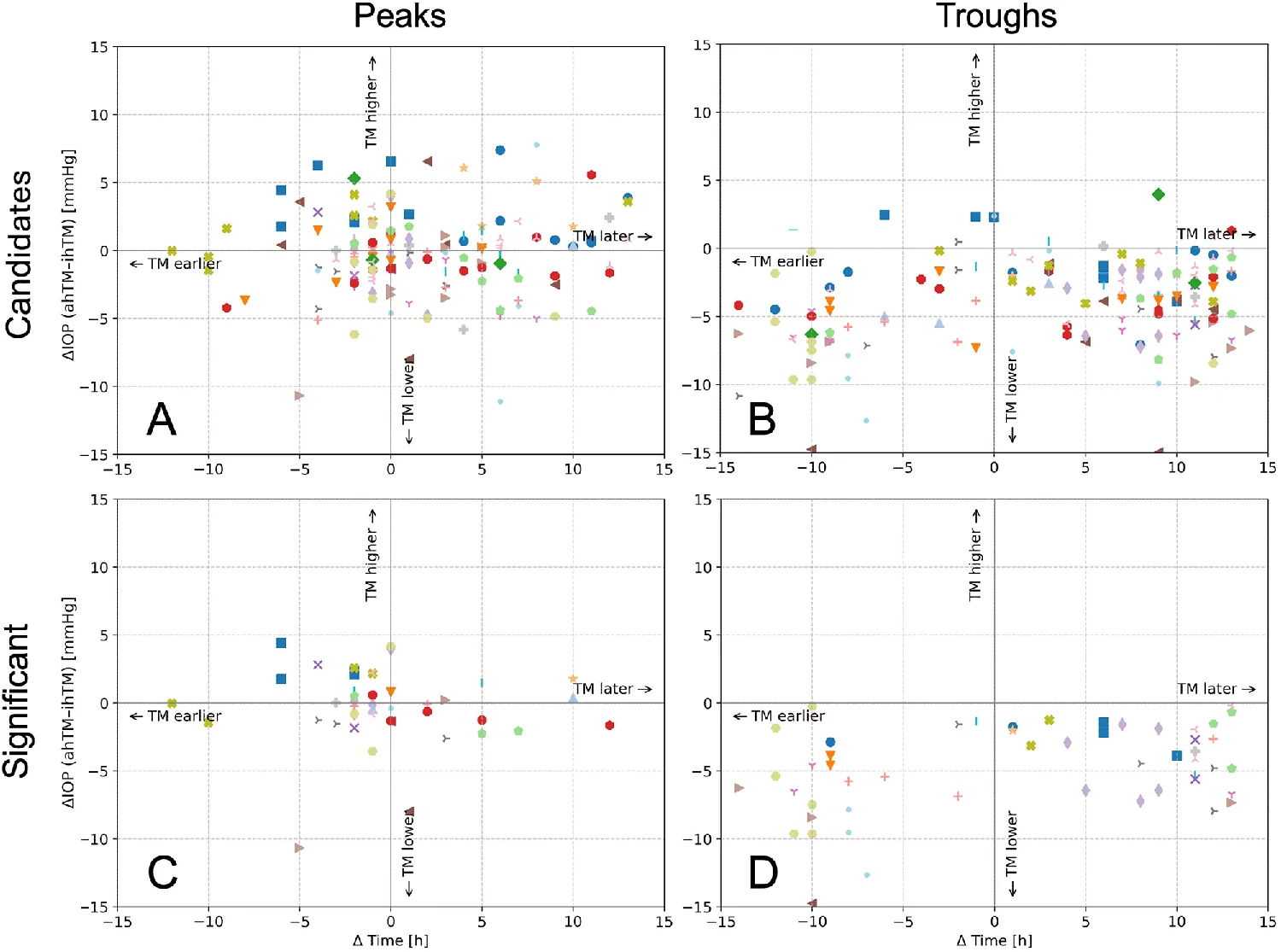
Scatterplots illustrating time- and IOP-related deviations of candidate and significant extreme hourly averaged at-home telemetric IOP measurements (ahTM), recorded during the 30 days preceding each visit, from in-hospital telemetric measurements (ihTM). (**A**) Candidate peaks (*n* = 154), (**B**) candidate troughs (*n* = 154), (**C**) statistically significant peaks (*n* = 45), and (**D**) statistically significant troughs (*n* = 54), each relative to the corresponding ihTM. Each symbol represents an individual (candidate or significant) extreme IOP value relative to its matched ihTM reference (origin). The *x*-axis indicates the Δ*t* between the telemetric extreme (peak or trough) and the ihTM (hours), and the *y-*axis indicates the ΔIOP between the at-home and in-hospital telemetric measurements (mm Hg).

Among the statistically significant troughs, 30 occurred after GAT measurements (Δ*t* = 8.8 ± 3.8 hours). Of these, 29 were associated with lower telemetric IOP than GAT-measured IOP (ΔIOP = –5.0 ± 2.4 mm Hg) and one with higher (ΔIOP = 0.8 mm Hg). A total of 24 troughs occurred before GAT (Δ*t* = –9.0 ± 3.6 hours); 21 were lower (ΔIOP = –6.4 ± 3.5 mm Hg), and three were higher (ΔIOP = 1.2 ± 1.2 mm Hg) than the GAT-measured IOP (Fig. 5D). Equivalent analyses performed with the simultaneous in-hospital telemetric measurements showed comparable distributions (Fig. 6; Table 2).

**Table 2.** Distribution of Statistically Significant Extreme At-Home Telemetric IOP Measurements (Peaks and Troughs) Relative to In-Hospital Measurements (GAT and Simultaneous EyeMate-SC Readings)

	In-Hospital GAT	In-Hospital EyeMate-SC Tonometry
Peak
Significant, *n*	45	45
Absolute Δtime (h)	3.33	3.33
Absolute ΔIOP (mm Hg)	1.84	2.68
Trough
Significant, *n*	54	54
Absolute Δtime (h)	8.91	8.91
Absolute ΔIOP (mm Hg)	4.9	5.28

The number of significant extremes, their mean absolute time difference (Δtime) from the corresponding in-hospital measurements based on nyctohemeral alignment, and the mean absolute IOP difference (ΔIOP) between the in-hospital reference values and extreme at-home telemetric measurements are shown.

## Discussion

The aim of this study was to investigate the diagnostic value of one-time GAT IOP measurements in OAG eyes after NPGS. The results demonstrated that single GAT measurements frequently fail to represent the IOP profile of the preceding 30 days.

In roughly 75% of cases, GAT measurements were located outside the IQR of telemetric measurements obtained during the same TOD over the preceding 30 days. In addition, a relevant number of peaks and troughs were identified in the telemetric dataset. Most peaks occurred within 3 hours before GAT measurements on nyctohemeral alignment. Troughs were observed both before and after GAT measurements, but with larger nyctohemeral misalignment to GAT.

These findings support two principal conclusions: First, GAT measurements poorly reflect the average IOP of the preceding 30-day period, even when accounting for TOD; second, clinically relevant extremes, including peaks and troughs, may remain entirely undetected in a successfully NPGS-operated patient population. This underscores the limited representativeness of infrequent measurements and highlights the dynamic nature of IOP beyond nyctohemeral fluctuations.

Importantly, these findings do not devalue GAT as a measurement method. In agreement with previous reports,^25^^–^^27^ we observed good agreement between GAT and telemetric IOP measurements when performed at the same time points, and results were comparable when contrasting 30-day data with in-hospital telemetric and GAT measurements. Rather, the limitation lies in the temporal sparsity of the measurements, as a single value provides only a snapshot of a highly dynamic parameter.

In clinical practice, GAT measurements are often implicitly interpreted as representative of IOP control over weeks to months. The present data indicate that this assumption might often be incorrect. The observed ΔIOP values, although seemingly small, may be clinically decisive for treatment adaptation, particularly in well-controlled patients with low average IOP values, where relative deviations have greater impact.

In patient populations with poorer IOP control, the limitations of infrequent GAT measurements are likely to be even more pronounced, as increased variability is typically associated with higher IOP levels. In such settings, fluctuations are more substantial and thus supposed to be even more difficult to capture with single measurements. This aligns with the findings of Downs and colleagues, who demonstrated in animal models that IOP can fluctuate by up to 10 mm Hg from day to day, emphasizing that snapshot measurements may not adequately capture the true dynamic character of IOP, as the authors stated.^7^^,^^8^^,^^11^

Moreover, the present analysis was restricted to the 30 days before a clinic visit. In routine clinical care, however, only four to six visits per year are typical. Given that IOP is known to fluctuate not only over days but also across weeks and months,^31^^,^^32^ extending the observation window to 60 or 90 days would theoretically further reduce the representativeness of a single GAT measurement.

In most cases, GAT measurements were higher than the IQR of the at-home telemetric IOP measurements, whereas only a minority was lower. One possible explanation for this asymmetry is a stress-related “white coat effect”. Supporting this hypothesis, Keren et al. showed that mental stress increased IOP by 1 mm Hg in patients with OAG, with greater effects in more advanced disease.^33^ Similarly, Barrow et al.^34^ reported higher IOP in dogs in the hospital than at home. In a study evaluating IOP responses during anesthetic induction procedures in non-human primates, Turner et al.^35^ found that IOP increased by 10% within 10 seconds of the technician entering the anteroom and peaked within about 1 minute of the animals first anticipating human contact, further underscoring the influence of stress-related stimuli on IOP.

Linear regression analysis further indicated that GAT tends to overestimate the average IOP at lower values and underestimate it at higher values, although the magnitude of this effect was small. This finding is clinically relevant as long as infrequent IOP measurements remain standard practice, particularly in situations where treatment adequacy is uncertain based on moderate or elevated GAT values. Consistent with this observation, previous studies have reported reduced precision of GAT at higher IOP levels, often resulting in falsely low readings.^36^^–^^38^ For this subanalysis, *z*-score normalization was applied to standardize the ΔIOP within each patient by centering it around the patient-specific mean and scaling it by the patient-specific SD. This transformation changes the unit of measurement from mm Hg to SD but preserves all individual observations without excluding outliers, expressing each value relative to the patient's own distribution.

Although GAT is a precise method, it is influenced by several factors, including corneal biomechanics, astigmatism, and operator variability.^38^^–^^42^ For example, intraoperator LoA for GAT have been reported to be –4.44 to 4.24 mm Hg, and interoperator LoA –2.78 to 3.78 mm Hg.^42^ In contrast, EyeMate-SC measurements are relatively independent of these factors. Animal experiments have demonstrated good agreement between EyeMate-SC readings and direct manometric measurements, with LoA of –2.6 to 3.2 mm Hg.^43^ While the design of the EyeMate-SC should ensure effective transchoroidal measurement, these agreement data support that the device measures the true IOP rather than the suprachoroidal space pressure, which may be up to 4 mm Hg lower than the IOP, as suggested by Emi et al.^44^ However, definitive validation in humans remains challenging.

Overall, our findings highlight the need for improved IOP monitoring strategies. Rebound tonometry enables patient self-measurement and provides more frequent data, potentially improving the estimation of average IOP and detection of fluctuations.^45^ Telemetric implants offer additional advantages by enabling high-frequency, longitudinal monitoring with minimal user dependency.^24^ The EyeMate-SC, designed for implantation during NPGS, provides near-continuous access to IOP data and is a promising tool for individualized glaucoma management.^24^^,^^25^ A recent analysis even indicated that short-term IOP metrics derived from the EyeMate-SC can be used to predict long-term IOP fluctuations with the aid of machine learning.^46^

The present study cohort consisted exclusively of NPGS patients, reflecting the approval context of the device.^25^ Although implantation requires a surgical procedure, it does not involve anterior chamber penetration. In contrast to contact lens–based sensors, the EyeMate-SC enables permanent IOP monitoring.^17^ The findings suggest that implantation may offer diagnostic advantages and could potentially be considered beyond combined surgical settings in high-risk patients.

Several limitations must be acknowledged. The study population was small, reflecting the early clinical evaluation phase of the device and precluding identification of contributing factors; therefore, larger studies are required.^25^ TOD analysis was limited by unequal data distribution, with underrepresentation of early-morning measurements and imbalance between time intervals. Although in-hospital GAT and telemetric measurements were compared with at-home telemetric measurements within the same TOD categories, circadian influences cannot be fully excluded. However, this distribution largely reflects routine clinical practice: At-home telemetric measurements were obtained continuously over 24 hours with a natural clustering during awake periods (Fig. 1), whereas in-hospital GAT measurements were predominantly performed during consultation hours (mostly between 9 AM and 3 PM) (Table 1). Thus, although this imbalance may limit circadian generalizability, it also mirrors the practical conditions under which GAT-based decision-making is routinely performed in clinical care. Accordingly, our findings should be interpreted in the context of these inherent sampling constraints, unless more resource-intensive 24-hour monitoring is employed.

Also, although the telemetric measurements obtained in this study do not represent continuous monitoring, they still provide substantially higher temporal resolution than routine GAT assessments. Current device iterations (not yet in use) enabling near-continuous measurement—for example, through integration into wearable systems—may further enhance data quality. Notably, IOP is also influenced by multiple physiological and environmental factors, including body position, physical activity, and seasonal variation.^47^^–^^49^ Finally, a single GAT measurement may coincide with a transient peak or trough independent of the TOD, further limiting its representativeness.

## Conclusions

This study demonstrates that, in successfully NPGS-operated patients, infrequent GAT measurements often fail to reflect the IOP profile of the preceding month and may miss clinically relevant extremes. Although GAT remains a reliable and precise measurement technique, its diagnostic value is limited when used at low frequency. These findings support a paradigm shift from infrequent snapshot measurements toward high-frequency monitoring strategies. Given the increasing burden of glaucoma, aging populations, and constrained healthcare resources, improved monitoring approaches are essential. Telemetric IOP monitoring represents a promising solution to enhance disease understanding and optimize individualized treatment.

## Supplementary Material

Supplement 1
