# Supplementary material for: What Do Infrequent Goldmann Applanation Tonometry Measurements Reveal About Intraocular Pressure Profiles After Nonpenetrating Glaucoma Surgery?
Source: Transl Vis Sci Technol. 2026 Aug 27;15(8):28. doi: 10.1167/tvst.15.8.28 (PMC13533311; doi:10.1167/tvst.15.8.28)
Supplement: Supplementary file 1 [file tvst-15-8-28_s001.pdf]

Supplementary Table 1. Averaged Goldmann applanation tonometry (GAT) intraocular pressure (IOP) measurements for each patient at each study visit.

Patient	D90 (mmHg)	D180 (mmHg)	D270 (mmHg)	D360 (mmHg)	D540 (mmHg)	D720 (mmHg)	D920 (mmHg)	D1080 (mmHg)
**1**	17.3	13.7	14.0	14.7	12.5			
**2**	4.3	5.3	8.0	10.0	12.0	14.0	11.7	10.7
**3**	5.3							
**4**	9.3	13.0	14.0	17.0	13.8	13.8	14.7	
**5**	14.0	14.0	17.3	12.7	12.0	15.7	12.7	
**6**	9.0	16.0	17.3	14.0				
**7**	18.3	15.0	16.0	16.0	16.0	15.3	16.7	
**8**	19.3	18.3	13.7	13.3				
**9**	13.0	15.7	17.0	16.3	16.7	14.7	21.7	15.7
**10**	8.7	9.0	6.7	10.0	9.0	5.3	9.7	7.3
**11**	13.0	14.0						
**12**	12.3	14.0	14.7	16.0	15.7	17.7	15.3	14.7
**13**	12.0	11.7	12.0					
**14**	20.0	22.0	20.3	12.3	18.0	16.0	22.3	20.3
**15**	13.0	13.0	11.3	12.3	14.0	15.0	19.0	
**16**	11.0	8.7	10.0	9.7	10.0	9.0	9.7	9.5
**17**	14.0	8.7	8.7	11.3	5.0	3.5	9.3	12.0
**18**	20.7	15.0	21.0	20.7	16.7	15.0	12.0	13.3
**19**	10.7	8.0	12.0	8.0	9.0	11.0	12.0	10.0
**20**	15.5	13.5	14.0	12.7	12.7	16.3	17.0	13.7
**21**	14.5	13.0	16.7	15.5	18.0	16.7	12.0	12.0
**22**	13.0	14.0	13.7					
**23**	14.0	16.5	18.0	18.0	18.0	17.0	19.0	17.0
**24**	11.5	12.5	14.0	14.5	14.5	13.5	15.0	13.5

Supplementary Table 2. Averaged in-hospital telemetric intraocular pressure (IOP) measurements for each patient at each study visit.

Patient	D90 (mmHg)	D180 (mmHg)	D270 (mmHg)	D360 (mmHg)	D540 (mmHg)	D720 (mmHg)	D920 (mmHg)	D1080 (mmHg)
**1**	17.7	9.6	9.9	12.3	10.0			
**2**	5.3	5.2	6.2	9.2	11.4	11.3	10.4	8.6
**3**	7.3							
**4**	11.1	11.9	13.5	16.0	18.5	6.8	8.7	
**5**	12.6	13.3	13.4	12.6	13.6	15.6	13.3	
**6**	12.0	14.0	15.0	13.2				
**7**	18.5	10.6	13.5	12.9	11.2	9.3	12.7	
**8**	16.0	15.4	14.6	11.5				
**9**	4.8	10.3	8.9	8.9	7.8	16.5	17.9	16.4
**10**	6.6	7.1	8.0	8.5	7.3	6.6	6.3	6.8
**11**	12.0	10.4						
**12**	10.5	8.3	12.5	11.3	10.9	12.7	11.6	13.6
**13**	17.5	13.8	10.4					
**14**	19.3	17.6	17.5	14.0	15.3	14.4	12.6	9.0
**15**	10.4	10.3	10.3	11.3	7.7	8.5	8.9	
**16**	8.1	8.1	6.8	8.0	9.6	10.3	9.5	10.5
**17**	11.8	5.3	4.5	7.9	8.5	6.5	8.4	12.4
**18**	14.0	15.9	19.0	17.6	14.7	12.4	9.3	8.3
**19**	9.7	8.6	9.2	10.2	10.0	10.3	10.0	9.2
**20**	11.6	10.8	10.1	9.4	7.5	6.9	7.2	11.6
**21**	16.0	14.5	14.2	16.2	16.7	15.8	13.2	13.2
**22**	18.8	11.5	11.4					
**23**	18.5	17.0	11.0	12.1	11.6	11.7	11.2	11.5
**24**	11.0	9.7	9.3	9.6	9.8	9.7	9.2	9.8

Supplementary Table 3. Differences in IOP (ΔIOP; EyeMate-SC – GAT) between in-hospital telemetric and Goldmann applanation tonometry measurements for each patient at each study visit.

Patient	D90 (mmHg)	D180 (mmHg)	D270 (mmHg)	D360 (mmHg)	D540 (mmHg)	D720 (mmHg)	D920 (mmHg)	D1080 (mmHg)
**1**	0.4	–4.1	–4.1	–2.4	–2.5			
**2**	1.0	–0.1	–1.8	–0.8	–0.6	–2.7	–1.3	–2.0
**3**	2.0							
**4**	1.8	–1.1	–0.5	–1.0	4.7	–6.9	–6.0	
**5**	–1.4	–0.7	–4.0	–0.1	1.6	–0.1	0.7	
**6**	3.0	–2.0	–2.4	–0.8				
**7**	0.1	–4.4	–2.5	–3.1	–4.8	–6.0	–4.0	
**8**	–3.3	–2.9	0.9	–1.8				
**9**	–8.2	–5.3	–8.1	–7.4	–8.9	1.9	–3.8	0.7
**10**	–2.1	–1.9	1.4	–1.5	–1.7	1.3	–3.4	–0.5
**11**	–1.0	–3.6						
**12**	–1.8	–5.7	–2.2	–4.7	–4.7	–4.9	–3.8	–1.1
**13**	5.5	2.1	–1.6					
**14**	–0.7	–4.4	–2.8	1.6	–2.7	–1.6	–9.8	–11.3
**15**	–2.6	–2.7	–1.0	–1.1	–6.3	–6.5	–10.1	
**16**	–2.9	–0.6	–3.2	–1.7	–0.4	1.3	–0.1	1.0
**17**	–2.2	–3.4	–4.1	–3.5	3.5	3.0	–1.0	0.4
**18**	–6.7	0.9	–2.0	–3.1	–1.9	–2.6	–2.7	–5.1
**19**	–1.0	0.6	–2.8	2.2	1.0	–0.7	–2.0	–0.8
**20**	–3.9	–2.7	–3.9	–3.3	–5.1	–9.4	–9.8	–2.1
**21**	1.5	1.5	–2.5	0.7	–1.3	–0.9	1.2	1.2
**22**	5.8	–2.5	–2.3					
**23**	4.5	0.5	–7.0	–5.9	–6.4	–5.3	–7.8	–5.5
**24**	–0.5	–2.8	–4.7	–4.9	–4.7	–3.8	–5.8	–3.7

Supplementary Table 4. Z-score-normalized IOP differences (ΔIOP; EyeMate-SC – GAT) between in-hospital telemetric and Goldmann applanation tonometry measurements for each patient at each study visit.

Patient	D90 (a.u.)	D180 (a.u.)	D270 (a.u.)	D360 (a.u.)	D540 (a.u.)	D720 (a.u.)	D920 (a.u.)	D1080 (a.u.)
**1**	1.6	–0.8	–0.9	0.1	0.0			
**2**	1.7	0.8	–0.6	0.2	0.4	–1.4	–0.2	–0.8
**3**								
**4**	0.8	0.0	0.2	0.1	1.5	–1.4	–1.2	
**5**	–0.4	–0.1	–1.9	0.3	1.2	0.3	0.7	
**6**	1.4	–0.6	–0.7	–0.1				
**7**	1.9	–0.4	0.5	0.2	–0.6	–1.3	–0.2	
**8**	–0.8	–0.6	1.4	0.0				
**9**	–0.8	–0.1	–0.8	–0.6	–1.0	1.6	0.3	1.3
**10**	–0.6	–0.5	1.5	–0.3	–0.4	1.4	–1.4	0.3
**11**	0.7	–0.7						
**12**	1.1	–1.2	0.9	–0.6	–0.7	–0.8	–0.1	1.5
**13**	1.0	0.0	–1.0					
**14**	0.7	–0.1	0.3	1.3	0.3	0.5	–1.3	–1.7
**15**	0.5	0.5	1.0	1.0	–0.6	–0.6	–1.7	
**16**	–1.3	0.1	–1.4	–0.5	0.3	1.3	0.4	1.1
**17**	–0.4	–0.8	–1.1	–0.9	1.5	1.3	0.0	0.4
**18**	–1.7	1.7	0.4	–0.1	0.4	0.1	0.1	–1.0
**19**	–0.3	0.6	–1.4	1.6	0.9	–0.2	–1.0	–0.2
**20**	0.4	0.8	0.4	0.6	0.0	–1.5	–1.6	1.0
**21**	0.9	0.9	–1.7	0.3	–1.0	–0.7	0.7	0.7
**22**	1.2	–0.6	–0.5					
**23**	2.0	1.1	–0.7	–0.4	–0.5	–0.3	–0.9	–0.3
**24**	2.1	0.7	–0.5	–0.6	–0.5	0.0	–1.2	0.1
